# The complete mitochondrial genome sequence and phylogenetic position of *Xylota coquilletti* (Hervé-Bazin 1914) (Diptera: Syrphidae: Eristalinae: Xylotini)

**DOI:** 10.1080/23802359.2022.2107955

**Published:** 2022-08-10

**Authors:** Zelin Pan, Xingfa Ci, Renwen Zheng, Qingfeng Tang, Sihan Lu

**Affiliations:** College of Plant Protection, Anhui Province Key Laboratory of Integrated Pest Management on Crops, Key Laboratory of Biology and Sustainable Management of Plant Diseases and Pests of Anhui Higher Education Institutes, Anhui Agricultural University, Hefei, China

**Keywords:** Mitochondrial genome, phylogeny, Eristalinae, Xylotini, *Xylota coquilletti*

## Abstract

The complete mitochondrial genome sequence of the *Xylota coquilletti* (Diptera: Syrphidae: Eristalinae: Xylotini) was sequenced and reported for the first time. The whole genome was 15,920 bp in length with the 37 classical eukaryotic mitochondrial genes and a control region. The nucleotide composition was included by 40.5% A, 39.6% T, 11.7% C, and 8.2% G, meaning that A + T (80.1%) was much greater than C + G (19.9%). It consisted of 22 transfer RNA genes (tRNAs), two ribosomal RNA genes (rRNAs), 13 protein-coding genes (PCGs), and a control region (CR). Phylogenetic analyses were performed using 13 PCGs and it was found that *Xylota coquilletti* was sister to *Ferdinadea cupera*. All this information could complement the mitochondrial data for a new tribe of Eristalinae.

*Xylota coquilletti* (Hervé-Bazin 1914) belongs to the tribe Xylotini (Diptera: Syrphidae: Eristalinae). Eristalinae includes nine tribes: Brachyopini, Callicerini, Cerioidini, Eristalini, Merodontini, Milesiini, Rhingiini, Sericomyiini, and Volucellini (Mengual 2015; Young et al. 2016). To date, the number of sequenced and reported complete mitochondrial genomes was only 25, belonging to species of Eristalini, Milesiini, Volucellini, Brachyopini, and Rhingiini. In this study, a complete mitogenome from the representative individual named *Xylota coquilletti* belonging to Xylotini was obtained to further identify the phylogenetic relationships in the subfamily.

The sequenced species were collected from Sijihuahai Park, Hefei City, Anhui Province, China (117°16′E, 31°08′N) on 11 June 2021. The vouchered individual was immediately stored in absolute ethanol and was frozen at −20 °C in Laboratory 1043, College of Plant Protection, Anhui Agricultural University (voucher number LSH-5mL-Aj13, Zelin Pan, panzelin333@sina.com). The total genomic DNA was extracted from the complete individual except for its genitalia. The mitogenome was sequenced on the MGI T7 platform with 150 bp paired-end reads and yielded 4.30 GB paired raw reads. The quality of the data was checked by FastQC (Andrews 2016). A total of 4.10 GB of clean paired-end reads (Phred scores >20) were quality-trimmed and were assembled using NOVOPlasty4.3.1 (Dierckxsens et al. 2017) with default parameters and the mitochondrial genome of *Xylota sylvarum* (Huo et al. 2018) (GenBank no. LR999962) used as a reference. Gene annotation was carried out by Geneious 8.1.3 (Kearse et al. 2012). PCGs were determined as open reading frames, rRNAs and tRNAs were identified using MITOS (Bernt et al. 2013).

The whole length of the mitochondrial genome from the *Xylota coquilletti* (GenBank no. MZ905457) was 15,920 bp, which included 40.5% A, 39.6% T, 11.7% C, and 8.2% G. In other words, the percentage of A + T (80.1%) was much greater than C + G (19.9%), revealing that the genome exhibited significant A/T bias. The mitochondrial genome was made of 22 transfer RNA genes (tRNAs), two ribosomal RNA genes (rRNAs), 13 protein-coding genes (PCGs), and a control region (CR). Most PCGs regarded the ATN putative as the start codon (five ATG, four ATT, and one ATA) except COXI, nad1, and nad5. They were started with CGA, TTG, and GTG, respectively. Besides, most PCGs regarded TAA as the termination codon except nad5, which was stopped with incomplete termination codon T. For tRNA genes, the length of the 22 genes was about 63–72 bp. The secondary structure of tRNA was a typical cloverleaf structure, except trnD and trnS1. In terms of two rRNA genes, the 16S rRNA was 1339 bp, which was placed between trnL1 and trnV. The 12S rRNA was placed between trnV and the CR with a length of 786 bp. The length of CR was 1016 bp and it was placed between 12SrRNA and trnI. According to the whole genome, there was no gene rearrangement.

In order to estimate the phylogenetic position of *X. coquilletti*, the mitochondrial DNA sequences of the other 28 Syrphidae species (25 Eristalinae as ingroup and three Syrphinae as outgroup) were selected from the GenBank database to further define the relationship within Eristalinae. All the PCGs of the 29 species data were aligned by multiple alignments using MAFFT 7 plugin in PhyloSuite 1.2.2 (Zhang et al. 2020). The gaps and the ambiguous sites were removed using the Gblocks plugin in PhyloSuite 1.2.2 (Zhang et al. 2020). The aligned PCGs data of the different species were concatenated using concatenate sequence plugin in PhyloSuite 1.2.2 (Zhang et al. 2020) and the best-fit substitution model for phylogenetic analyses was determined using PartitionFinder 2.7 plugin in PhyloSuite 1.2.2 (Zhang et al. 2020). The maximum-likelihood tree was established by MEGA X (Kumar et al. 2018) based on 13 PCGs (Figure 1). It showed that Eristalinae could be divided into six major clades: Eristalini, Milesiini, Volucellini, Chrysogasterini, Cheilosiini, and Xylotini. Eristalinae was monophyletic, which was consistent with the previous report (2003). Besides, each tribe was also monophyletic. It is revealed in Figure 1 that *X. coquilletti* (Xylotini) should be regarded as a sister to *Ferdinadea cupera* (Rhingiini). In this research, essential fundamental data to understand the phylogenetic relationships among major lineages of the subfamily was provided.

**Figure 1.**
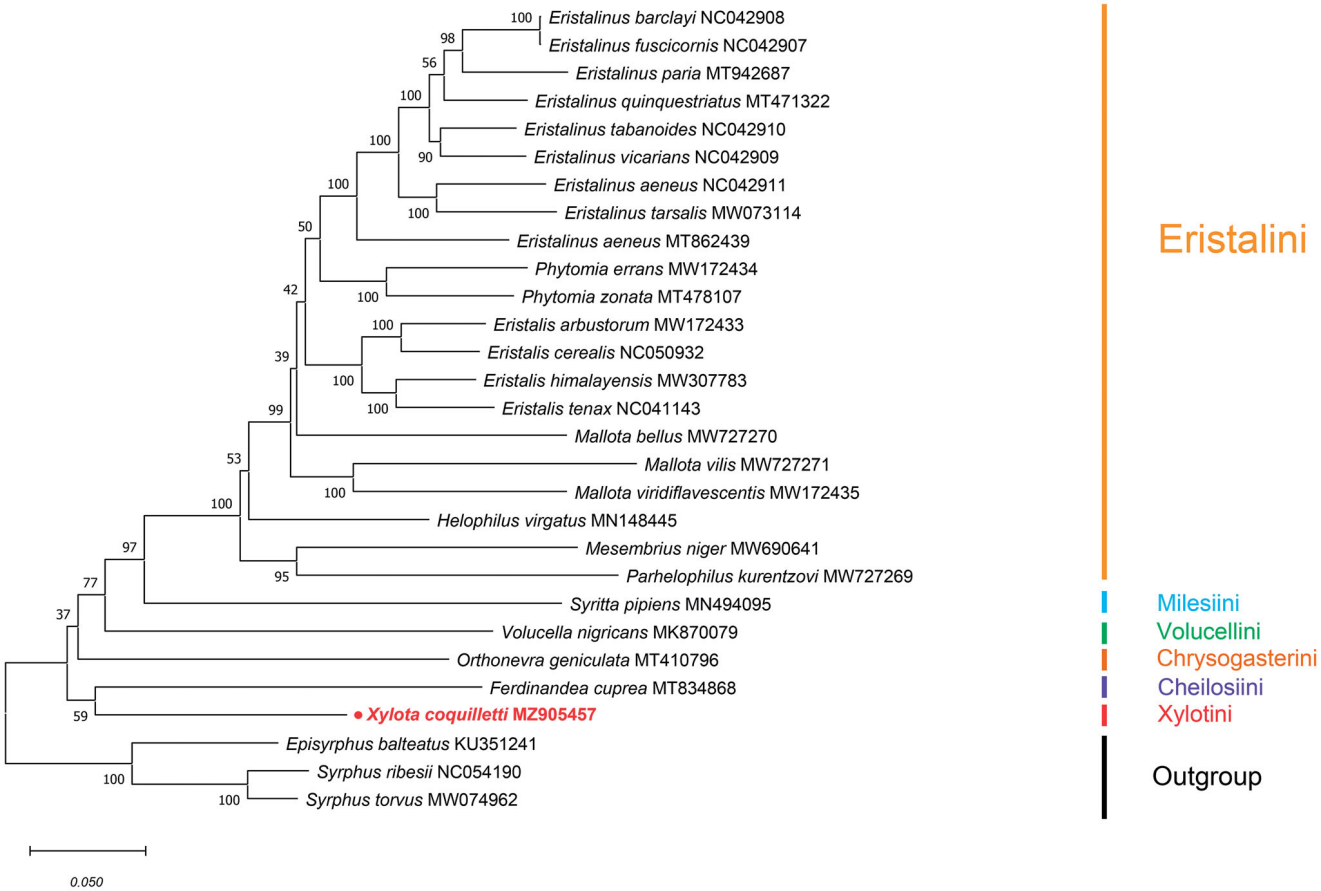
ML consensus tree for *Xylota coquilletti* and Eristalinae based on concatenated 13 PCGs using maximum-likelihood (ML). Numbers at the nodes represent bootstrap support values based on 1000 replicates and indicate the new sequence in this study.

## Data Availability

The genome sequence data that support the findings of this study are openly available in GenBank of NCBI at https://www.ncbi.nlm.nih.gov/ under the accession no. MZ905457. The associated BioProject, Bio-Sample numbers, and SRA are PRJNA757554, SAMN21018174, and SRS9990438, respectively.
